# Amoxicillin degradation ability of *Bacillus cereus* C1 isolated from catfish pond sludge in Vietnam

**DOI:** 10.1016/j.heliyon.2022.e11688

**Published:** 2022-11-19

**Authors:** Tam-Anh Duong-Nguyen, Minh Hoang Pham, Nghi Hue Lam, Cuong Quoc Pham, Trung Duc Le, Bao Minh Tran, Tung Van Tra

**Affiliations:** a Faculty of Biology and Biotechnology, University of Science, Vietnam National University Ho Chi Minh City (VNU-HCMC), 227 Nguyen Van Cu, Dist. 5, Ho Chi Minh City, Viet Nam; b Institute for Environment and Resources – IER, Vietnam National University Ho Chi Minh City (VNU-HCMC), 142 To Hien Thanh, Dist. 10, Ho Chi Minh City, Viet Nam

**Keywords:** *Bacillus cereus*, Antibiotic resistance, Amoxicillin degradation

## Abstract

The biological removal of antibiotic residue in the environment has earned great interest. This study presented the biodegradation of amoxicillin (AMX) using B. cereus C1 isolated from the catfish pond sludge in Vietnam. This AMX-degrading bacterial strain grew well in the range of temperatures between 25ΟC and 40ΟC under aerobic condition. In a culture medium containing nitrogen source of NH4Cl (1 g.L−1) alone, the bacterium showed a AMX degradation ability of 54%. The AMX degradation ability of this bacterial strain was the highest level of 94% in the culture medium with 1.5 g.L−1 of NH4Cl and 3 g.L−1 of glucose. B. cereus C1 exhibited a great antibiotic degradation capability on high AMX concentration of 250 μg.mL−1 of AMX with AMX removal efficiency of 84% in 16 h of cultivation.

## 1 Introduction

The main source of the antibiotic residue in the environment is from the fragmentary metabolization of antibiotics by human and animal. Others come from hospital and veterinary waste, industrial antibiotic producing company, waste from ordinary animal feeds, and agricultural farm where plant biomass is increased by using antibiotics (Rajilić-Stojanović et al., 2012). Of the antibiotics waste, amoxicillin (AMX) is one of the most typical antibiotics found in the environmental pollutants (De La Torre et al., 2012). AMX belongs to the β-lactam group of antibiotic drugs. This kind of antibiotics discomposes the cell walls of the bacteria in their growth process (Baghapour et al., 2014). AMX, unlike other natural antibiotic in penicillin family, is stable in acid condition and difficult to be hydrolyzed (Yang et al., 2020). The present of AMX in the environment may accompany to the increase of the antibiotic resistant bacteria (Gavrilescu et al., 2015).

Physical and chemical methods have been proposed for removing antibiotic residue in the environment, such as ultraviolet (UV) - and solar based methods (UV/H_2_O_2_, solar/H_2_O_2_), ozonation, Fe^2+^ or Fe^3+^/H_2_O_2_ photocatalysis, or photocatalysis by TiO_2_ (Michael-Kordatou et al., 2018). Nonetheless, the main hindrance of these techniques is their high cost, thus they are inaccessible to developing countries.

An alternative for those expensive techniques is the microbial degradation technologies. They are inexpensive and more ecofriendly than chemical and physical methods. In the metabolic processes of microorganisms, numerous enzymes with high catalytic activity are generated that can modify the structure of antibiotic directly or indirectly, which can lower or inactivate the antibiotic activity (Li and Zhang, 2010). Moreover, the products of these biological processes are very simple such as water, CO_2_, nitrogen, and simple organic compound. There are many reports on biological degradation of antibiotics in the environment. *Acinetobacter, Stenetrophomonas maltophilia* and Aeromonas veronii, which are found in Ireland and England, can tolerate AMX in freshwater (Huys et al., 2000). The strains of *Flavobacterium* isolated from marine sediments of Uranouchi Bay, Japan can corrupt a group of antibiotics including AMX (Maki et al., 2006). Escherichia coli has also been reported the AMX resistant activity (Grundt et al., 2012). Many studies on β-lactam antibiotic resistance of Bacillus cereus have also been reported (Fiedler et al., 2019; Kim et al., 2015; Luna et al., 2007; Park et al., 2009; Yim et al., 2015). This bacterial strain could completely remove AMX in the day 14 of incubation process (Liyanage and Manage, 2016).

This paper evaluated the AMX degradation ability of B. cereus C1 isolated in a catfish pond sludge in Vietnam by examining the cell growth and the AMX degeneration ability of this strain in different supplementary sources of carbon and nitrogen, and evaluating the ability of the strain to decompose the antibiotic at different AMX concentrations.

## 2 Materials and methods

### 2.1 Sludge sample and culture media

The sludge of the catfish pond in Chau Thanh District, An Giang Province, Vietnam was collected and dried at the room temperature until reaching a moisture of 11%. The sludge was then stored at 4 °C in a refrigerator.

The Luria-Bertani (LB) broth (Sezonov et al., 2007) was used in this experiment, containing: NaCl 10 g.L^−1^, peptone 10 g.L^−1^, yeast extract 5 g.L^−1^, and AMX 50 μg.mL−1. The medium was adjusted to pH 7 using 1N of NaOH. The LB-agar medium used to isolate the bacteria contained 2% of agar and 50 μg.mL−1 of AMX (Bio-Amox LA, Bio-Pharmachemie, Viet Nam).

The LB media were prepared with different supplementary sources of carbon and nitrogen at different concentrations to identify the growth and the AMX degradation ability of the isolated strain. The carbon sources used for the investigations were glucose (0.5 g.L−1 – 7 g.L−1), starch (0.5 g.L−1 – 1.5 g.L−1), and condensed molasse (0.5 g.L−1 – 1.5 g.L−1). The nitrogen sources were KNO3 (0.1 g.L−1 – 2 g.L−1), NH4Cl (0.1 g.L−1 – 2 g.L−1), and urea (0.1 g.L−1 – 7 g.L−1).

### 2.2 Experimental methods

#### 2.2.1 Isolation of amoxicillin – degrading bacterial strain

The amoxicillin – degrading bacterium were isolated from the slugde in one of catfish ponds in Chau Thanh District, An Giang Province, Vietnam because the slugde often contains high antibiotic residues derived from the empirical and uncontrolled use of antibiotics to treat fish disease. 1 g of the sludge was heated at 80ΟC in 10 min to eliminate the non-spore forming bacteria. The pretreated sludge was then cooled down to room temperature and vortexed with 20 mL of deionized water for 20 min. Then, the mixture was kept stable for 15 min to settle all the sediment. Next, 100 μL of the mixture was spread on the LB-agar medium containing 50 μg.mL−1 of AMX. After 24 h of incubation at room temperature, the colonies were picked and screened AMX degradation ability. The isolate was stored at 4ΟC for further investigation.

#### 2.2.2 Identification and phylogenetic analysis

The 16S rDNA was extracted and amplified from the genomic DNA of the isolate using the procedure showed in the literature (Nguyen et al., 2018). The 27f (5′-AGAGTTTGATCMTGGCTCAG-3′) and 1492r (5′-TACGGYTACCTTGTTACGACTT-3′) primers were used in this procedure. Then, the PCR products of 16S rDNA of both strands were sequenced using a cycle sequencing system. The 16S rDNA sequences were then compared with the reference sequences in NCBI gene bank using the Basic Local Alignment Search Tool (BLAST). The most similar reference sequences were collected and used to create the phylogenetic tree by the Neighbor-joining method using the MEGA-X software version 10.0.5.

#### 2.2.3 Determination of the growth curve of the isolated bacterium

The isolate was cultivated in the LB liquid medium containing 50 μg mL^−1^ of AMX for determining the growth curve. The inoculum level of the culture was fixed at the OD600 value of 0.3. The growth curve of the isolate was evaluated by measuring the optical density at 600 nm (OD600) of the bacterial culture using a spectrophotometer (UV-5100, METASH, China) (Ignatova et al., 2009; Altuntas et al., 2010) in 24 h of cultivation at different temperatures (50^Ο^C, 45^Ο^C, 40^Ο^C, and room temperature). The growth curve of the isolate was then represented as the plot of the cell density vs. the culture time.

#### 2.2.4 AMX degradation ability determination

The AMX concentration in the culture medium was determined using the spectrophotometric method (Li and Yang, 2007). After 16 h of cultivation, the culture medium was centrifuged at 6000 rpm to eliminate the cells. Then, 1 mL of the supernatant was placed in a flask with 2 mL of 0.2% of Folin's reagent and 2 mL of K_2_HPO_4_–KH_2_PO_4_ buffer solution of pH 9 added sequentially. Next, the solution was diluted to 12.5 mL with deionized water, mixed well and stood for 50 min at room temperature. After that, the solution was measured the optical density at 468nm (OD468) against the blank which was the pure LB medium prepared with the same procedure.

The AMX concentration remained in the culture media after 16 h of cultivation was then estimated by Eq. (1):

(1) y=0.0015x×d

where,

*x* is the standard AMX concentration (μg),

y is the optical density at 468nm (OD468)

*d* is the dilution factor.

The AMX degradation ability was calculated by Eq. (2):

(2) $$DA\;\left(\%\right)=\frac{A_{i}-A_{r}}{A_{i}}\times 100$$

where,

DA is the AMX degradation ability (%)

*A*_*i*_ is the initial amount of AMX in the culture medium (μg)

$A_{r}$ is the remaining amount of AMX in the culture medium after 16 h of cultivation (μg)

#### 2.2.5 Statistical analyses

The data of these experiments were presented as Mean ± Standard deviation of at least three replications. Groups comparison was done by two-way ANOVA followed by Tukey's post hoc test using GraphPad Prism software version 9. Statistically significant differences of the data were considered at p < 0.05.

## 3 Results and discussion

### 3.1 The 16S rDNA gene identification and phylogenetic tree of the isolated bacterium

**Figure 1:**
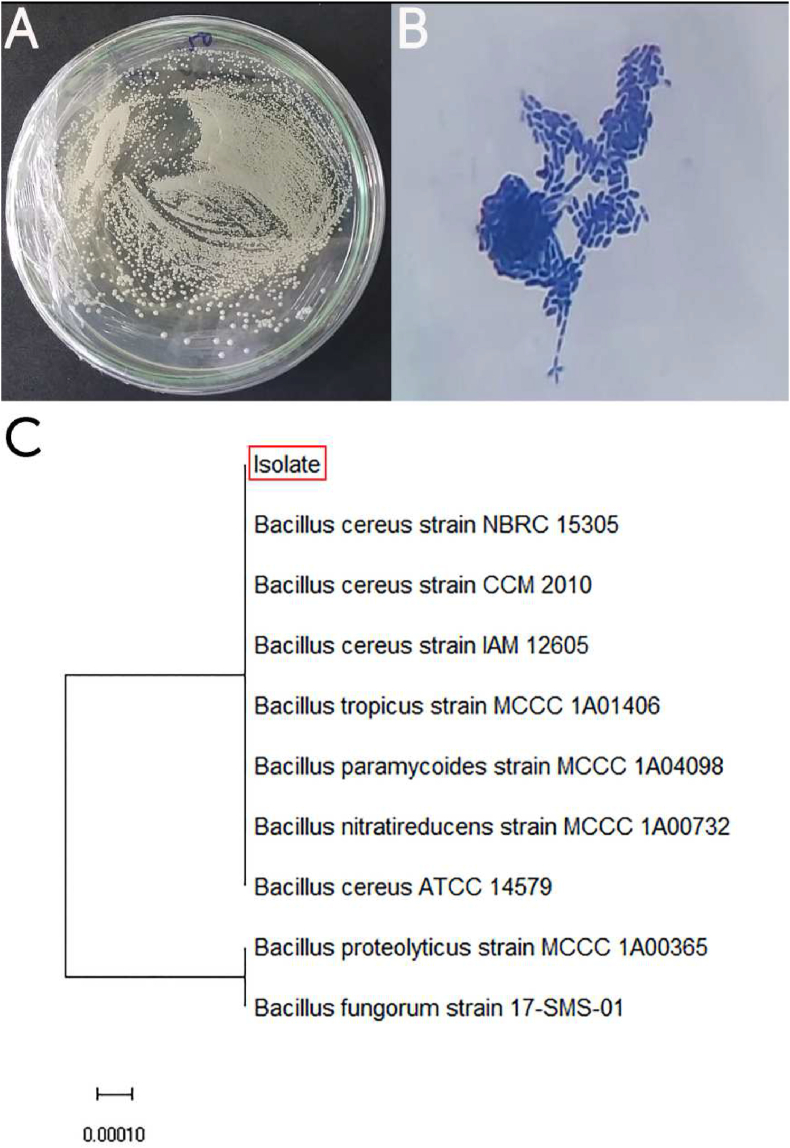
A. Morphology of isolated colony, **B**. Gram result of the isolated bacteria (×800), and **C**. The phylogenetic analysis of the 16S rDNA genes of the strain. The tree was established using a neighbor-joining method with 1000 boot strappings. The scale bar represents 0.0001 replacement per nucleotide position.

After 24 h of incubation, there was only one kind of colony appear on the Petri dish. The isolated colony had a milky white color, round shape, and smooth surface (Figure 1A). Microscopic image of this colony shows that it was a Gram positive, rode shape bacterial strain (Figure 1B). From the phylogenetic analysis based on the 16S rDNA sequence, shown in Figure 1C, the isolated AMX - degrading bacterium was named *Bacillus cereus* C1 because it showed a similarity of 100% to *Bacillus cereus*. *B. cereus* presents in nearly everywhere in nature, from decaying matters to vegetables and various kinds of food, from fresh to salt waters (Bottone, 2010; Park et al., 2009) and it is also reported that strains of *B. cereus* can resist to the β-lactam family of antibiotic such as penicillin, oxacillin, and cephalosporins (Park et al., 2009).

### 3.2 Growth curve of the *B. cereus C1* strain

**Figure 2:**
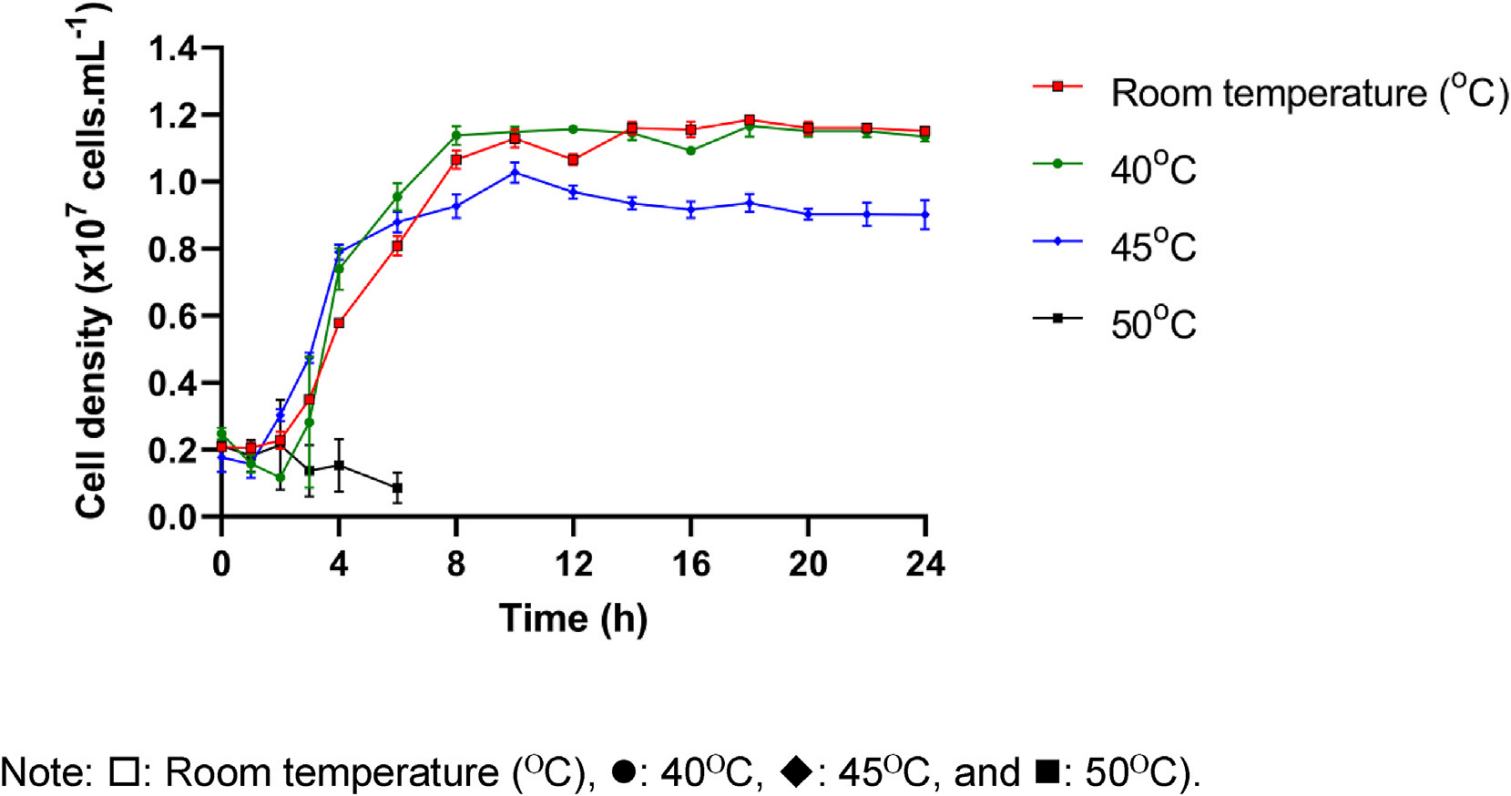
Growth curve of B. cereus C1 at different temperatures.

Figure 2 demonstrates the growth curve of the *B. cereus* C1 cultivated in LB medium containing 50 μg.mL−1 of AMX at different temperatures including room temperature (25 ΟC – 32 ΟC), 40ΟC, 45ΟC, and 50ΟC. The growth of the B. cereus C1 at 50ΟC was negative from (2.114 ± 0.143) × 106 cells. mL−1 in the initial stage of cultivation to (8.560 ± 4.530) × 105 cells. mL−1 at 6 h of cultivation, which indicated that this strain could not survive at the temperature higher than 50ΟC. The growth at other temperatures follows an S-shape growth curve. This bacterium showed a lag phase of 2–3 h, a short logarithmic phase of around 5 h and a prolonged stationary phase of 16h in the cultivation time of 24 h.

The highest growth occurred nearly the same at room temperature and at 40ΟC with cell density of (1.156 ± 0.024) × 107 cells. mL−1 at 16 h at room temperature and (1.157 ± 0.013) × 107 cells. mL−1 at 12 h at 40ΟC. Therefore, the optimum temperature for this strain is between room temperature and 40ΟC. The bacterial strain was cultured at room temperature for the following experiments.

### 3.3 AMX degradation ability of B. cereus C1 in different supplementary sources of carbon and nitrogen

**Figure 3:**
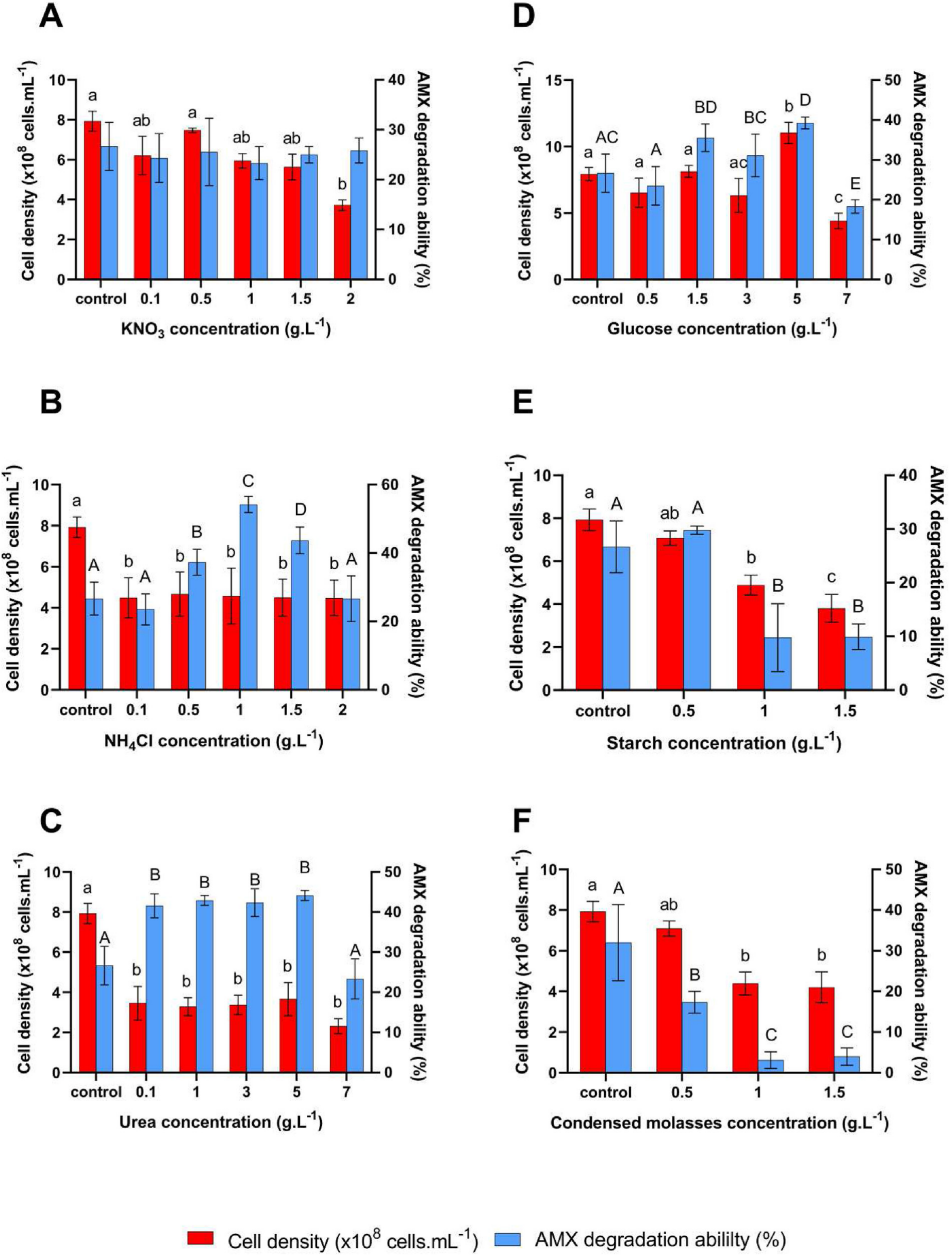
B. cereus C1 cell density (× 108 cells. mL−1) and amoxicillin (AMX) degradation ability (%) of the strain at different concentration of carbon and nitrogen sources supplemented to the culture medium (g.L−1). A. KNO3, B. NH4Cl, C. Urea, D. Glucose, E. Starch, and F. Condensed molasses (CM). Control means the LB medium containing 50 μg.mL−1 of AMX. Statistical analysis by two-way ANOVA followed by Tukey's post hoc test. Different letters show significant differences between data groups (p < 0.05). Lower-case letters represent the comparison of the cell density group, and upper-case letters represent the comparison of the AMX degradation group. Values represent the Mean ± SD of 3 replica.

Different carbon and nitrogen sources were exerted to the culture medium at different concentrations to examine the growth and the AMX decomposition ability of B. cereus C1. The results were shown in Figure 3.

Regarding the nitrogen sources, the growth of the bacterium in the medium supplemented with KNO3 was nearly the same as in the LB medium (the control one) (Figure 3A). Moreover, the increase in the KNO3 concentration resulted in the decrease of the bacterial growth. The AMX degradation ability of B. cereus C1 grew at various concentrations of KNO3 from 0.1 g.L−1 to 2 g.L−1 showed no significant difference from the control of approximately 27% (p > 0.05). It was likely that KNO3 had no effect on the growth and the AMX degradation ability of B. cereus C1 strain. Ammonium chloride (NH_4_Cl) exerting to the medium decreased the cell growth from (8.014 ± 0.458) × 108 cells. mL−1 (control) to (4.263 ± 1.059) × 108 cells. mL−1 at 1 g.L−1 of NH4Cl. This strain was reported to be lack of ability to utilize the *NH*_4_^+^ ion for growth (Mols and Abee, 2008). However, in our study, it was found that NH4Cl at increasing concentrations in the culture medium increased AMX degradation of B. cereus C1, from 26.67% in the control one to roughly 54% at 1 g.L−1 of NH4Cl (Figure 3B), even though this inorganic nitrogen source was used with the aim to reduce the cost of culture media. The β-lactamase could be one of possible explanations for the ability to degrade AMX of B. cereus C1 (Fiedler et al., 2019). NH4Cl as cheap inorganic nitrogen source perhaps had reinforcing effects on the biosynthesis or the activity of β-lactamase produced by B. cereus C1. The same phenomenon happened for urea. Urea was likely to enhance the degradation of the AMX in the culture medium as well. The AMX degradation of roughly 44% was obtained (Figure 3C). This strain was reported to have the ability to hydrolyze of urea into *NH*_4_^+^ by producing urease (Mols and Abee, 2008). This ion, in turn, induced the activity of the β-lactamase and increases the AMX degradation ability of the strain. However, adding urea to the culture medium decreased the cell growth, which dropped from (8.014 ± 0.458) × 108 cells. mL−1 in the control one to (3.659 ± 0.826) × 108 cells. mL−1 at all of the urea concentrations.

In case of carbon sources, glucose expressed to be the most suitable for the growth and the AMX degradation activity as well (Figure 3D). At 5 g.L−1 of glucose concentration, the maximum growth and AMX degradation ability were obtained, which were (1.099 ± 0.101) × 109 cells. mL−1 and roughly 40%, respectively. It was found that 5 g.L-1 of glucose was the optimal glucose level for B. cereus C1 growing and degrading AMX. The glucose concentration higher than this level such as 7 g.L-1 glucose showed an inhibitory effect on growth as well as AMX degradation capability of this bacterial strain. The inhibitory effect of higher glucose concentration could be related to the harmful osmotic pressure or decrease in glucose adsorption of the bacterium (Dike et al., 2014; Shukor et al., 2009). Both starch and condensed molasse did not support a good growth as well as the AMX degradation ability for this bacterial strain (Figure 3E, F). The starch was exerted at a concentration higher than 0.5 g.L−1, the growth and the AMX degradation ability decreased dramatically, from (8.014 ± 0.458) × 108 cells. mL−1 and 29% to (3.808 ± 0.645) × 108 cells. mL−1 and 10% when adding from 0.5 g.L−1 to 1.5 g.L−1 of starch, respectively (Figure 3E). The results indicated that this bacterium could not produce amylases which are enzymes used for starch hydrolysis into simple and consumable sugars. Adding condensed molasse to the culture medium also decreased the growth and the AMX degradation ability of B. cereus C1 from 26.67% to roughly 3% comparing to the control medium (Figure 3F). It could be explained that condensed molasse could contain large number of heavy metals such as iron, zinc, copper, calcium, etc. which could prohibit the cell growth and inhibit the enzyme activity (Roukas, 1998), resulting in the low cell density and AMX degradation ability as well.

Table 1 summary the maximum cell density and AMX degradation ability at different carbon and nitrogen sources. Based on the table, NH_4_Cl and glucose will be selected to examine the concerted effect on the degradation of AMX.

**Table 1:** Comparison of different carbon and nitrogen sources on the growth (cells.mL−1) and the AMX degradation ability (%).

Source	Cell density (x108 cells.mL^−^1)	AMX degradation ability (%)
KNO3 (5g.L^−^1)	7.464 ± 0.163a	27.500 ± 8.25A
NH4Cl (1g.L^−^1)	4.263 ± 1.059b	54.222 ± 2.341B
Urea (5g.L^−^1)	3.659 ± 0.826b	44.153 ± 1.243CD
Glucose (5g.L^−^1)	10.992 ± 1.014c	39.259 ± 1.481D
Starch (0.5g.L^−^1)	7.077 ± 0.334a	29.778 ± 0.77AD
CM (0.5g.L^−^1)	7.101 ± 0.369a	17.333 ± 2.667AD

Note: The selected concentration of each source is based primarily on the highest AMX degradation ability and followed by the cell density. Statistical analysis by two-way ANOVA followed by Tukey's post hoc test. Different letters show significant differences between data groups (p < 0.05). Lower-case letters represent the comparison of the cell density group, and upper-case letters represent the comparison of the AMX degradation group. Values represent the Mean ± SD of 3 replica.

### 3.4 AMX degradation ability of *B. cereus C1* in a combination of NH_4_Cl and glucose

Different concentrations of NH_4_Cl (0.5, 1, 1.5 g.L−1) and glucose (1.5, 3, 5 g.L−1) were selected to evaluate the concerted effect on the growth and AMX degradation ability of *B. cereus* C1.

**Figure 4:**
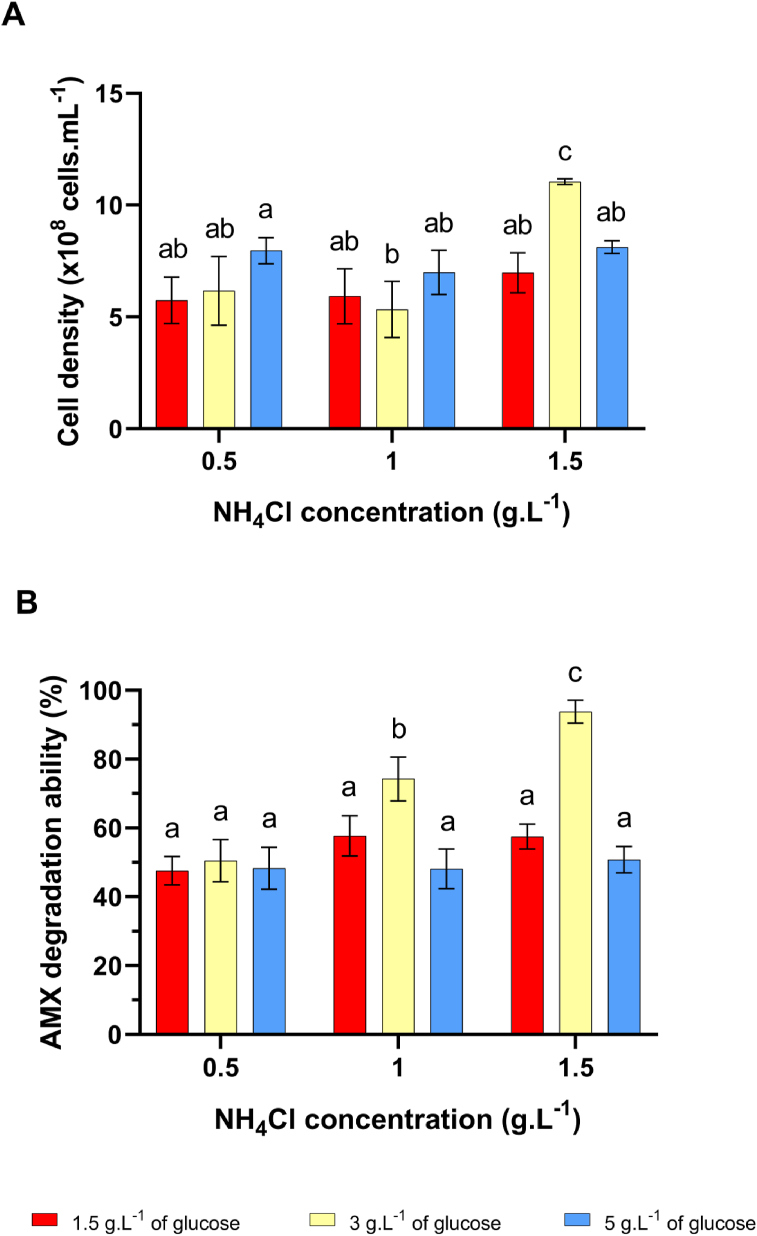
B. cereus C1 cell density and AMX degradation ability of the strain at different concentrations of NH4Cl and glucose (g.L−1). A. Cell density (× 108 cells. mL−1), B. AMX degradation ability (%). Statistical analysis by two-way ANOVA followed by Tukey's post hoc test. Different letters show significant differences between data groups (p < 0.05). Values represent the Mean ± SD of 3 replica.

An impressively increasing effect was observed as adding both NH_4_Cl and glucose to the culture medium (Figure 4). Although the growth was the same (1.099 ± 0.101) × 109 cells. mL-1 (in the culture medium containing only 5 g.L−1 of glucose) and (1.104 ± 0.013) × 109 cells. mL-1 (in the culture medium containing 1.5 g.L−1 of NH4Cl and 3 g.L−1 of glucose) (Figure 4A), the AMX degradation capability of the bacterium increased 1.74 times, from 54% (in the culture medium containing only 1 g.L−1 of NH4Cl) to nearly 94% (in the culture medium containing 1.5 g.L−1 of NH4Cl and 3 g.L−1 of glucose) (Figure 4B). Thus, there could be an additional effect of NH4Cl and glucose as added the culture medium simultaneously. NH4Cl induced the β-lactamase activity, and glucose enhanced the growth of the cell. Combining NH4Cl and glucose influence increasing the growth and activity of the β-lactamase in B. cereus C1, which increased the AMX degradation ability of the strain.

### 3.5 AMX degradation ability of B. cereus C1 at different AMX concentrations

**Figure 5:**
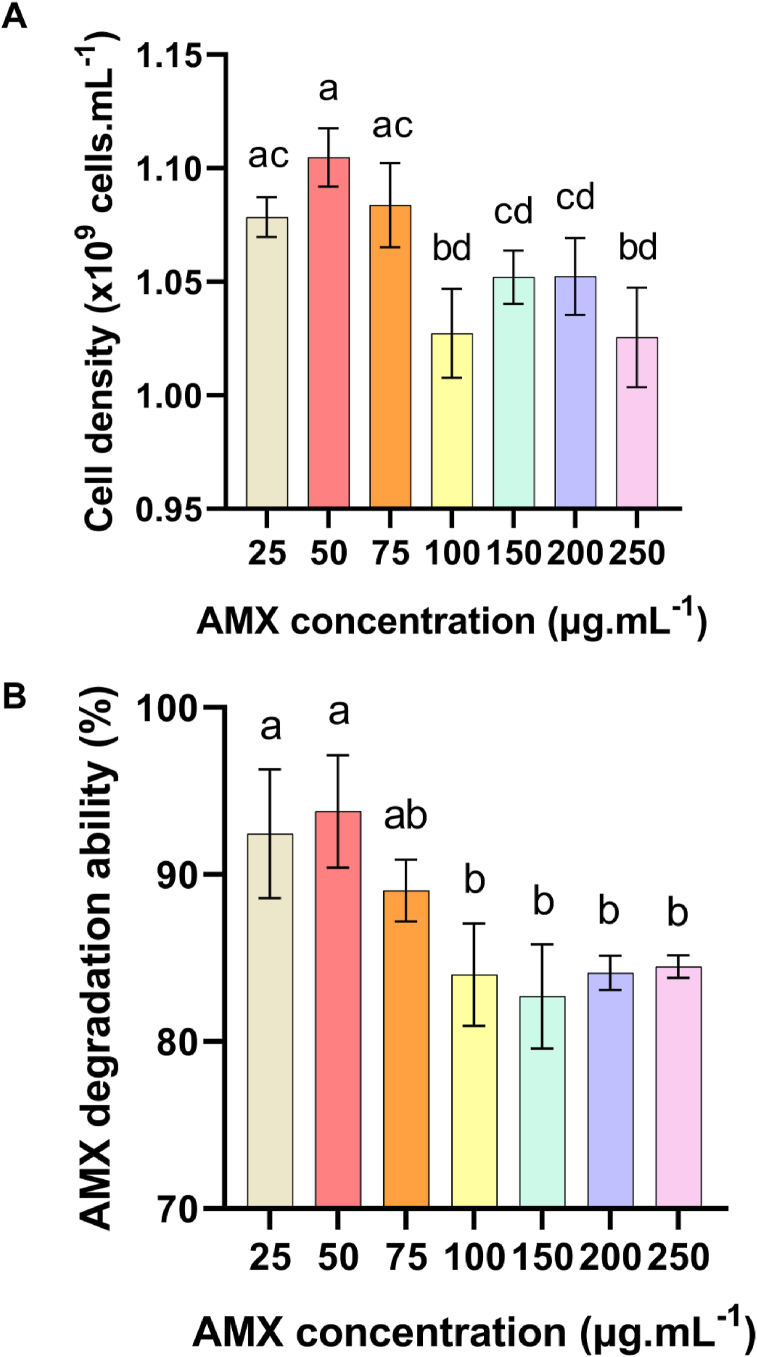
B. cereus C1 cell density and AMX degradation ability of the strain at different concentrations of AMX (μg.mL−1). A. Cell density (× 108 cells. mL−1), B. AMX degradation ability (%). Statistical analysis by one-way ANOVA followed by Tukey's post hoc test. Different letters show significant differences between data groups (p < 0.05). Values represent the Mean ± SD of 3 replica.

The AMX degradation ability of the strain was further evaluated in the culture medium containing increasing AMX concentrations ranging from 25 μg.mL−1 to 250 μg.mL−1 with 1.5 g.L−1 of NH4Cl and 3 g.L−1 of glucose (Figure 5). The maximum cell growth and the AMX degradation ability were achieved at the AMX concentrations increased from 25 μg.mL−1 to 75 μg.mL−1 (Figure 5A). At the AMX concentration higher than 75 μg.mL−1, there was an inhibitory effect on the growth and a decrease in the degradation ability of the B. cereus C1 strain (Figure 5B). The inhibitory effect could be derived from the enzyme β-lactamase inhibition at high substrate concentration (AMX) (Huang and Penner, 1991; Penner and Liaw, 1994). Consequently, that inhibition declined the cell growth. However, B. cereus C1 strain. still grew in the culture medium containing very high AMX concentration, up to 250 μg.mL−1 with the cell density reaches (1.026 ± 0.022) × 109 cells. mL−1. Many studies reported that B. cereus could resist to β-lactam antibiotics by producing β-lactamase (Fiedler et al., 2019; Kim et al., 2015; Luna et al., 2007; Park et al., 2009; Yim et al., 2015). This study had confirmed the resistance of B. cereus to amoxicillin even at very high AMX concentration of 250 μg.mL−1 with the degradation ability of approximately 84% within 16 h of cultivation.

## 4 Conclusions

This study had assessed the growth and AMX degradation ability of the B. cereus C1 strain isolated from the catfish pond sludge in different carbon and nitrogen sources supplemented to the medium and assessed the degradation ability of AMX when increasing the AMX concentration as well. The bacterium had an optimum growth between room temperature and 40ΟC with the growth reached (1.156 ± 0.024) × 107 cells. mL−1 at 16 h at room temperature, and (1.157 ± 0.013) × 107 cells. mL−1 at 12 h at 40ΟC. Nitrogen sources showed the high AMX degradation ability of the strain, 1 g.L−1 of NH4Cl alone showed the highest degradation ability (54%). The highest cell growth ((1.099 ± 0.101) × 109 cells. mL−1) was obtained with 5 g.L−1 of glucose when used as a single element. When combining 1.5 g.L−1 of NH4Cl and 3 g.L−1 of glucose, the growth reached (1.104 ± 0.013) × 109 cells. mL−1 along with the high degradation ability (94%). B. cereus C1 also indicated the possibility to use for treating high AMX concentration, up to 250 μg.mL−1 of AMX, the degradation ability was roughly 84% in 16 h of cultivation. Thus, B. cereus C1 could be utilized as an effective biological tool for removing amoxicillin residue in the environment.

## Declarations

### Author contribution statement

Tam-Anh Duong-Nguyen: Conceived and designed the experiments; Performed the experiments; Analyzed and interpreted the data; Contributed reagents, materials, analysis tools or data; Wrote the paper.

Hoang-Minh Pham: Performed the experiments; Analyzed and interpreted the data; Wrote the paper.

Nghi Hue Lam: Performed the experiments.

Cuong Quoc Pham: Performed the experiments.

Trung Duc Le: Conceived and designed the experiments; Contributed reagents, materials, analysis tools or data; Wrote the paper.

Bao Minh Tran: Analyzed and interpreted the data; Contributed reagents, materials, analysis tools or data.

Tung Van Tra: Performed the experiments; Analyzed and interpreted the data.

### Funding statement

This research is funded by Vietnam National University HoChiMinh City (VNU-HCM) under grant number C2020-24-01.

### Data availability statement

Data included in article/supp. material/referenced in article.

### Declaration of interests statement

The authors declare no conflict of interest.

### Additional information

No additional information is available for this paper.
